# Oleuropein-Enriched Olive Leaf Extract Affects Calcium Dynamics and Impairs Viability of Malignant Mesothelioma Cells

**DOI:** 10.1155/2015/908493

**Published:** 2015-11-26

**Authors:** Carla Marchetti, Marco Clericuzio, Barbara Borghesi, Laura Cornara, Stefania Ribulla, Fabio Gosetti, Emilio Marengo, Bruno Burlando

**Affiliations:** ^1^Istituto di Biofisica, Consiglio Nazionale delle Ricerche, Via De Marini 6, 16149 Genova, Italy; ^2^Dipartimento di Scienze e Innovazione Tecnologica (DISIT), Università del Piemonte Orientale, Viale Teresa Michel 11, 15121 Alessandria, Italy; ^3^Dipartimento di Scienze della Terra dell'Ambiente e della Vita (DISTAV), Università degli Studi di Genova, Corso Europa 26, 16132 Genova, Italy

## Abstract

Malignant mesothelioma is a poor prognosis cancer in urgent need of alternative therapies. Oleuropein, the major phenolic of olive tree (*Olea europaea* L.), is believed to have therapeutic potentials for various diseases, including tumors. We obtained an oleuropein-enriched fraction, consisting of 60% w/w oleuropein, from olive leaves, and assessed its effects on intracellular Ca^2+^ and cell viability in mesothelioma cells. Effects of the oleuropein-enriched fraction on Ca^2+^ dynamics and cell viability were studied in the REN mesothelioma cell line, using fura-2 microspectrofluorimetry and MTT assay, respectively. Fura-2-loaded cells, transiently exposed to the oleuropein-enriched fraction, showed dose-dependent transient elevations of cytosolic Ca^2+^ concentration ([Ca^2+^]_i_). Application of standard oleuropein and hydroxytyrosol, and of the inhibitor of low-voltage T-type Ca^2+^ channels NNC-55-0396, suggested that the effect is mainly due to oleuropein acting through its hydroxytyrosol moiety on T-type Ca^2+^ channels. The oleuropein-enriched fraction and standard oleuropein displayed a significant antiproliferative effect, as measured on REN cells by MTT cell viability assay, with IC_50_ of 22 *μ*g/mL oleuropein. Data suggest that our oleuropein-enriched fraction from olive leaf extract could have pharmacological application in malignant mesothelioma anticancer therapy, possibly by targeting T-type Ca^2+^ channels and thereby dysregulating intracellular Ca^2+^ dynamics.

## 1. Introduction

Malignant mesothelioma is a poor prognosis cancer with worldwide increasing insurgence, arising from the pleura and other mesothelial tissue and showing close association with asbestos exposure. An effective therapeutic approach for the disease is currently lacking, while alternative treatments are urgently needed [1]. Among alternative remedies for cancer treatment, there is a growing interest in the anticancer action of natural substances, some of which are present in large amounts in byproducts from agrofood chains.

Olive (*Olea europaea* L.) is a main temperate fruit crop, mostly destined to oil production [2]. Olive oil is reputed to be an important health promoting factor in the Mediterranean diet, especially because of its alleged prevention of cardiovascular problems, metabolic syndrome, cancer, alleviation of inflammatory and autoimmune conditions, and wound healing [3]. However, olive tree leaves have a several-century-long tradition in the folk medicines of the Mediterranean basin and are currently contemplated in the Pharmacopoeia Ph. Eur. 5 [4].

Leaves and other materials from olive tree pruning accumulate in yearly amounts as high as about 25 kg per tree [5]. This material is generally disposed by olive tree growers, representing an overhead cost of olive and oil production, while the possibility of using it as biomass for thermal energy is under study [6]. Olive leaves are used in the form of herbal extracts, tea, and powder for nutritional supplement and cosmetics [4], but these products represent a minimal portion with respect to the huge amount of disposed materials.

The major leaf secondary metabolite is the secoiridoid oleuropein, a glycosylated ester of elenolic acid with hydroxytyrosol (2-(3,4-dihydroxyphenyl)ethanol). Oleuropein leaf content has been reported to vary about tenfold among different olive varieties, rating on average between 10 and 100 mg/g of dry matter [7, 8]. Many other biologically active phenolics are present in olive leaves, including hydroxytyrosol itself [9].

Oleuropein is deemed to have great potential as an antioxidant and food additive, but also as a possible therapeutic tool. A wide range of studies on oleuropein have been carried out using* in vitro* assays, animal models of disease, or human volunteers, in order to explore possible beneficial effects for human health [10]. The reported findings mainly include antioxidant and anti-inflammatory effects, hepatoprotection, neuroprotection, hypoglycemic and hypolipidemic activities, and cardiovascular protection [11, 12]. Antiproliferative activities on cancer cell lines, antitumor effects in animals, and antimicrobial and antiviral effects have also been shown [13].

Hydroxytyrosol in olive leaves, fruits, and extracts is believed to derive from oleuropein hydrolysis, both by specific enzymes and as an extraction artifact [9].* In vitro* and* in vivo* studies suggest that this compound shares remarkable antioxidant and anti-inflammatory power with oleuropein, allegedly related to antiatherogenic, antithrombotic, cardioprotective, and anticancer effects [14, 15].

The value of olive leaf extract for possible use in health products and medical food has been previously assessed in terms of antioxidant and antimicrobial power [16]. However, the profitable reutilization of agrofood chain byproducts needs to overcome several problems, including the costs of transport and processing and the seasonal availability of these materials. In this study, we have developed a rapid method for obtaining olive leaf extract enriched in oleuropein, involving raw extraction, extract partitioning, and chromatographic separation. The material has been tested in pharmacologically relevant, intracellular Ca^2+^ mobilization assay and antiproliferative assay on mesothelioma cells. In these experiments, standard oleuropein has been used as control, while hydroxytyrosol has been also used in Ca^2+^ assays to provide insight into the Ca^2+^ mobilizing properties of the oleuropein molecule.

## 2. Materials and Methods

### 2.1. Plant Material

Branch specimens of* Olea europaea* L. cultivar “Taggiasca” [17] were obtained from olive groves located at Imperia, Italy, owned by the Pietro Isnardi S.r.l. food company. The material was taxonomically identified by one of us (LC), and a voucher specimen was deposited at DISTAV, University of Genova (cat.: GE-*Olea europaea* cultivar “Taggiasca”). Leaves were separated from twigs, air-dried for one week, minced to <1 mm using a grinder, and then subjected to extraction procedure.

### 2.2. Reagents and Solutions

HPLC grade methanol Chromasolv (>99.9%) and glacial acetic acid were purchased from Sigma-Aldrich (Milwaukee, USA). Ammonium acetate (99%) was acquired from Fluka (Buchs, Switzerland). Ultrapure water was produced by a Millipore Milli-Q system (Milford, USA). Analytical grade chemicals were purchased from Sigma-Aldrich, unless otherwise specified.

For LC/MS analysis, standard stock solutions of oleuropein and hydroxytyrosol (each at concentration of 100.0 mg L^−1^) were prepared in methanol and diluted as required with a buffer solution of ammonium acetate 10.0 mM brought to pH 4.0 for acetic acid. For* in vitro* bioassays, stock solutions of oleuropein and hydroxytyrosol (each at 100 mM) were prepared in DMSO and diluted as indicated with cell medium or loading buffer for Ca^2+^ measurements. Stock solutions were preserved at −20°C in dark conditions and were stable for three months.

### 2.3. Olive Leaf Extraction Procedure

The minced leaves were extracted with a mixture of 2-propanol : water of 9 : 1. Aliquots of 10 g of leaves were extracted twice with 200 mL of solvent each time. The extraction was carried out at RT for about 1.5 h. The resulting solutions were mixed and the solvent was eliminated under vacuum, yielding an amount of 2.35 g of dried material. This raw extract was then partitioned between a mixture of methanol : water of 3 : 1 and a mixture of toluene : petroleum ether of 2 : 1 (100 mL each phase). The hydrocarbon phase became deep green, indicating that it contained all the chlorophyll of the raw extract. The aqueous methanol phase was dried under vacuum, yielding 1.8 g of subextract. This material was then again partitioned between water and 2-butanone (100 mL each phase), to remove sugars and other water-soluble compounds. The organic phase was dried out, obtaining a partitioned subextract that amounted to 1.03 g (43% recovery from the first raw extract, 10% from the plant material). A control TLC showed that in the former partition no oleuropein passed in the hydrocarbon phase, while in the latter only a tiny amount of it was lost in the water phase.

### 2.4. Chromatographic Separation

Liquid chromatography was performed by means of an MPLC system, consisting of an Alltech 426 HPLC pump equipped with a VWR LaPrep 3101 detector. Glass columns from Omnifit were used, home-packed with Fluka silica gel 100 RP-18 (15–35 *μ*m) stationary phase. TLC was performed with Merck F_254_ glass plates (RP-18); the spots were detected using a UV lamp (254 and 366 nm) and additionally by spraying with sulfovanillin and heating at 100°C, obtaining an oleuropein coral pink spot on TLC.

The above subextract was loaded on a RP-18 preparative column and eluted with 80% water and 20% methanol. Fractioning was monitored through the UV detector (*λ* = 250 nm) and with TLC. Finally, an oleuropein-enriched fraction was collected and subjected to further study.

### 2.5. LC/MS Analysis

#### 2.5.1. Apparatus

LC/MS analysis was performed on the above raw extract and subfractions by Nexera Liquid Chromatography Shimadzu (Kyoto, Japan) system equipped with a DGU-20A3R Degasser, two LC-30AD Pumps, a SIL-30AC Autosampler, a CTO-20AC column compartment, and a CMB-20A Lite system controller. The system was interfaced with a 3200 QTrap LC-MS/MS system (AB Sciex, Concord, Canada) by a Turbo V interface equipped with an ESI probe. The 3200 QTrap data were processed by Analyst 1.5.2 software (Toronto, Canada).

#### 2.5.2. UHPLC-MS/MS Conditions

The stationary phase was a Kinetex C18 column (2.1 mm × 100 mm, 2.6 *μ*m) (Phenomenex, Italy). The mobile phase was a mixture of ammonium acetate 10.0 mM in ultrapure water with the addition of 0.1% acetic acid (A), and aqueous ammonium acetate 10.0 mM/methanol 5/95 (v/v) with the addition of 0.1% acetic acid (B), eluting at flow-rate 0.400 mL min^−1^ in the following gradient conditions: 0.0–0.5 min 5% B, 0.6–8.0 min 65% B, 8.1–12.0 min 100% B, and 12.1–15.0 min 5% B. The injection volume was 5.0 *μ*L. Oven temperature was set at 40°C.

The turbo ion spray (TIS) ionization was obtained using the Turbo V interface working in negative ion mode. The instrumental parameters were set as follows: curtain gas (N_2_) at 30 psig, nebulizer gas GS1 and GS2 at 50 and 40 psig, respectively, desolvation temperature (TEM) at 550°C, collision activated dissociation gas (CAD) at 6 units of the arbitrary scale of the instrument, and ion spray voltage (IS) at −4500 V.

The 3200 QTrap was used in scheduled multiple reaction monitoring (sMRM) considering the transitions of each species at a prefixed retention time. Unit mass resolution was established and maintained in each mass-resolving quadrupole by keeping a full width at half maximum (FWHM) of about 0.7 u.

The analytes were previously subjected to MS/MS characterization study, to identify the fragmentation patterns taking place under increasing collisional energy. Characterization experiments were carried out for direct infusion of 1.0 mg L^−1^ standard solutions of each analyte connected through a T valve to the syringe pump (syringe flow-rate 20.0 *μ*L min^−1^; chromatographic pump flow-rate 200 *μ*L min^−1^). For each species, the most intense transition was used for the quantitative analysis and referred to as “quantifier” transition, while the second intense one (the “qualifier” transition) was employed in the identification step, as a confirmation. The “quantifier” and “qualifier” transitions and the instrumental potential values for each compound are reported in Table 1.

Calibration plots of five concentration levels (between LOQ value and 1000.0 *μ*g L^−1^), relating the peak area of the “quantifier” transition signal (*y*) versus standard concentration (*x*), were built for all the analytes. To avoid a possible influence of the experimental error on the execution order of analyses, the standard solutions were injected in randomized order. For all the analytes a linear regression fit was used with a weighting factor 1/*x* and for all the calibration plots a good linearity with regression coefficients (*R* ^2^) always greater than 0.9982 was obtained.

Detection limit values expressed as the concentration of the analyte that gives a signal equal to the average background (*S*_blank_) plus 3 times the standard deviation of the blank (LOD = *S*_blank_ + 3*s*_blank_) are 0.3 *μ*g L^−1^ for hydroxytyrosol and 0.4 *μ*g L^−1^ for oleuropein. The quantitation limits (LOQ), evaluated as LOQ = *S*_blank_ + 10*s*_blank_, are 1.0 *μ*g L^−1^ for hydroxytyrosol and 1.2 *μ*g L^−1^ for oleuropein.

### 2.6. *In Vitro* Cell Culture

*In vitro* experiments were carried out using the tumorigenic malignant mesothelioma REN cell line [1]. Cells were grown in DMEM, supplemented with 10% foetal bovine serum (FBS, Euroclone), at 37°C, in a 5% CO_2_, fully humidified atmosphere.

### 2.7. Cell Viability Assay

Cell viability was determined on REN cells by the MTT assay. Cells were settled in 96-well plates for 24 h, exposed to various agents for 48 h as specified, incubated with 100 *μ*L/mL tetrazolium salt (MTT, 5 mg/mL in PBS) in cell culture medium without serum for 3 h at 37°C, and treated with a solution of 1 N HCl-isopropanol (1 : 24, v/v) followed by mixing to dissolve the dark-blue formazan crystals formed. After a few minutes at room temperature, the plates were read at 550 nm in a VMax microplate reader (Molecular Devices, Sunnyvale, CA).

### 2.8. Intracellular Ca^2+^ Measurements

Cytosolic free Ca^2+^ concentration was measured in fura-2-loaded REN cells using a microspectrophotometry fluorescence-ratio setup equipped with a perfusion system, as previously described [18]. Cells were incubated in loading buffer containing 10 *μ*M fura-2-AM at 37°C for 30 minutes, washed with buffer, and mounted on the stage of an inverted microscope (Axiovert Zeiss, Germany), where they were continually superfused with different solutions. Cells were illuminated by a xenon lamp through a wavelength selector monochromator; emission was observed through ×40 quartz objective and recorded by a photomultiplier. The ratio *E*_340_/*E*_380_ was calculated every 40 msec to acquire a time-dependent internal Ca^2+^ sensitive signal.

At the end of each experiment, cells were incubated with 10 *μ*M ionomycin in a Ca-free solution containing 2 mM EGTA for 20−40 min until the ratio reached a minimum value (*R*_min_); then EGTA was washed out, normal external Ca^2+^ (1 mM) was restored, and the ratio readily reached a maximum value (*R*_max_). Finally, MnCl_2_ (5 mM) was added to the bath to quench the fura-2 fluorescence and determine the background (cell autofluorescence) values. The fluorescence emissions relative to each excitation wavelength (*E*_340_ and *E*_380_, resp.) were corrected for this background signal before ratio *R* = *E*_340_/*E*_380_ determination. Internal Ca^2+^ was calculated according to the Grynkiewicz equation [19].

### 2.9. Statistics

Statistics were obtained with the *R* package, version 3.0.1 (http://www.r-project.org/foundation/), using *t*-test with Bonferroni's correction for multiple comparisons. Cytotoxicity was determined using a logistic dose-response curve as reported in Ranzato et al. [20]. Analysis of data from Ca^2+^ measurements and determination of EC_50_ values were done by Sigmaplot 8 (Systat Software Inc., San Jose, CA).

## 3. Results

Multistep processing of olive leaves consisted of the following: (1) extraction of dried, minced leaves in 2-propanol : water (9 : 1), to obtain a raw extract; (2) partitioning between methanol : water (3 : 1) and toluene : petroleum ether (2 : 1), followed by partitioning of the dried aqueous methanol phase between water and 2-butanone, to obtain a subextract; (3) liquid (column) chromatography separation of the dried organic phase, to obtain an oleuropein-enriched fraction (see Methods). Oleuropein and hydroxytyrosol quantification in the raw extract and fractions was achieved by LC-MS technique. Data showed a progressive enrichment in oleuropein, up to a value of 60% w/w in the final fraction obtained through chromatographic separation (Table 2).

In order to evaluate the pharmacological potentials of the oleuropein-enriched, leaf extract fraction, and of its major component oleuropein, we tested the ability of mobilizing cell Ca^2+^ in mesothelioma REN cells. Intracellular Ca^2+^ is a main regulator of cell growth, and therefore agents able to deregulate Ca^2+^ dynamics can be possibly used to hinder cell proliferation.

**Figure 1:**
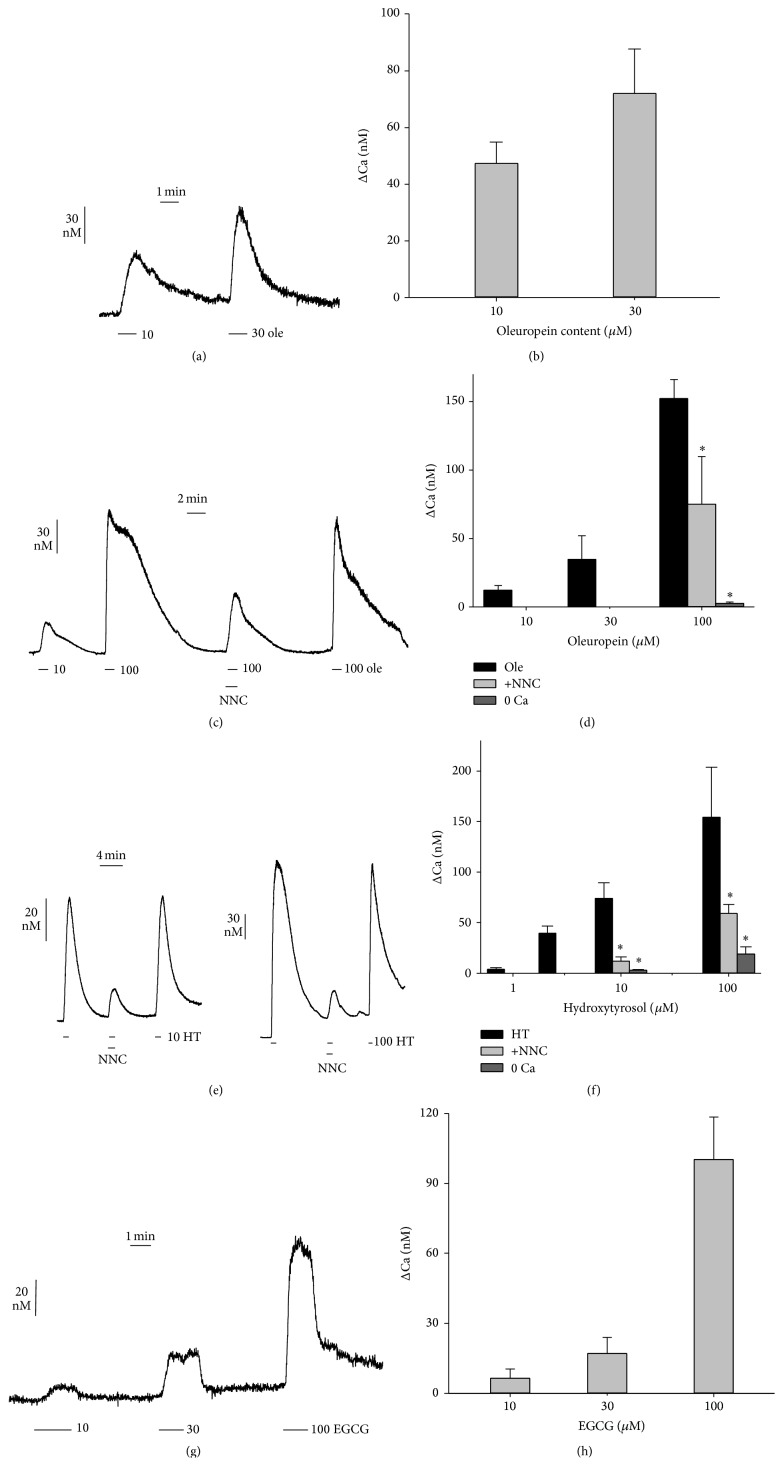
Time course of [Ca^2+^]_i_ level in mesothelioma REN cells loaded with fura-2 and alternatively exposed to the oleuropein-enriched fraction from olive leaf extract (a, b), to standard oleuropein (c, d) or to standard hydroxytyrosol (e, f). The oleuropein fraction and standard oleuropein have been used at nominal concentrations of oleuropein, expressed in *μ*moles/L. (a) Induction of [Ca^2+^]_i_ spikes in REN cells loaded with fura-2 by transient exposure to the oleuropein fraction (ole) at 10 and 30 *μ*M oleuropein. (b) Quantification of dose-dependent induction of [Ca^2+^]_i_ spikes by the oleuropein fraction. Data are means ± s.e.m. (*n* ≥ 3) of Δ[Ca^2+^]_i_ (difference between peak value and basal level of [Ca^2+^]_i_). (c) Induction of [Ca^2+^]_i_ spikes in REN cells by transient exposure to standard oleuropein (ole) at 10 and 100 *μ*M. The inhibitory effect of the T-type Ca^2+^ channel blocker NNC-55-0396 (5 *μ*M) and recovery after washout are shown. (d) Quantification of dose-dependent induction of [Ca^2+^]_i_ spikes by standard oleuropein (EC_50_ = 53 *μ*M), and inhibition by Ca^2+^-free medium (0 Ca) or NNC-55-0396 measured at 100 *μ*M oleuropein. Data as above; *∗* = *p* ≤ 0.01, *t* test. (e) Induction of [Ca^2+^]_i_ spikes by standard hydroxytyrosol (HT) at 10 and 100 *μ*M, inhibitory effect of NNC-55-0396, and recovery after washout. (f) Quantification of dose-dependent effect of hydroxytyrosol (EC_50_ = 12 *μ*M) and inhibition by Ca^2+^-free medium (0 Ca) or NNC-55-0396 measured at 10 and 100 *μ*M hydroxytyrosol. Data and statistics as above. (g, h) Induction of [Ca^2+^]_i_ spikes by EGCG and statistics of dose-dependent effect (EC_50_ = 69 *μ*M). Data as above.

Fura-2-loaded cells were transiently exposed by perfusion to the oleuropein fraction. This treatment induced [Ca^2+^]_i_ rises followed by a prompt recovery of the baseline level upon washout (Figure 1(a)). Data indicate that the observed [Ca^2+^]_i_ spikes are not due to cell membrane injury but rather depend on the influx of Ca^2+^ through membrane Ca^2+^ channels. Moreover, the [Ca^2+^]_i_ transient elevations were dependent on the nominal concentration of oleuropein (10–30 *μ*M) contained in the extract (Figure 1(b)).

To explore more in depth the mechanism of action, we also studied the Ca^2+^ mobilizing property of standard oleuropein. Similar to the oleuropein fraction, increasing doses of standard oleuropein, in the range 10–100 *μ*M, induced dose-dependent [Ca^2+^]_i_ spikes (Figures 1(c) and 1(d)). Removal of Ca^2+^ from the external solution almost abolished the oleuropein effect (Figure 1(d)). In addition, the use of the T-type Ca^2+^ channel inhibitor NNC-55-0396 (Sigma, ≥98%, HPLC) [21] reversibly reduced the Ca^2+^ spike induced by the highest dose of 100 *μ*M oleuropein (Figures 1(c) and 1(d)), suggesting at least a partial involvement of T-type Ca^2+^ channels.

We tried to provide some hint as to which oleuropein moiety could be active on cell Ca^2+^ mobilization. Hydroxytyrosol is known to induce modulatory effects on [Ca^2+^]_i_ [22]. We therefore used standard hydroxytyrosol on fura-2-loaded REN cells, observing the induction of dose-dependent [Ca^2+^]_i_ spikes that also in this case were inhibited by Ca^2+^-free medium. Moreover, NNC-55-0396 also hindered the hydroxytyrosol-induced [Ca^2+^]_i_ spikes, with 80% inhibition at 10 *μ*M hydroxytyrosol and 65% inhibition at 100 *μ*M hydroxytyrosol (Figures 1(e) and 1(f)).

As for comparative quantification of the effect on [Ca^2+^]_i_, dose-response data allowed estimating an EC_50_ of 12 *μ*M for hydroxytyrosol and of 53 *μ*M for oleuropein, showing that hydroxytyrosol is more powerful than oleuropein in inducing Ca^2+^ spikes. No established drug is known to induce T-type Ca^2+^ channel opening. We therefore used the natural phenolic epigallocatechin-3-gallate (EGCG) as positive control, since we had previously shown that this compound induces Ca^2+^ rise in REN cells via T-type Ca^2+^ channels [23]. Presently, EGCG was found to induce dose-dependent [Ca^2+^]_i_ spikes with an estimated EC_50_ of 69 *μ*M (Figures 1(e) and 1(f)).

**Figure 2:**
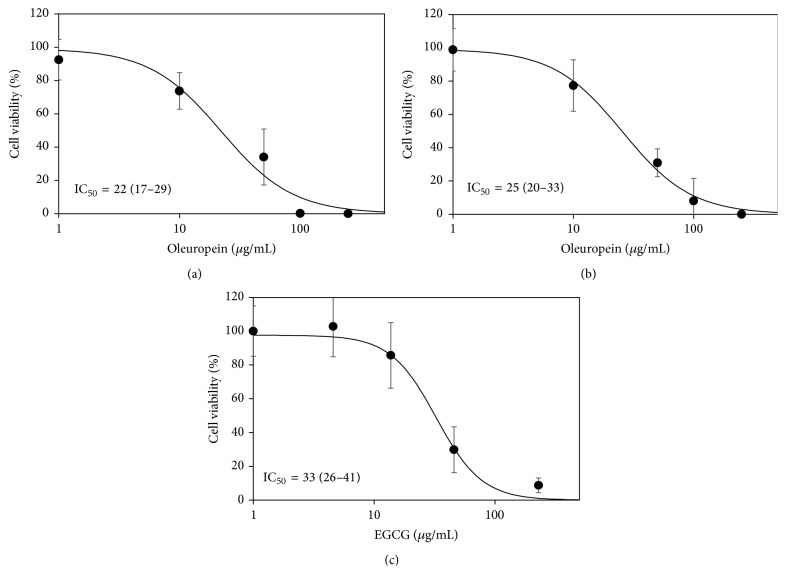
Dose-response data of cell viability obtained with the MTT assay after exposure of REN cells for 48 h to the oleuropein-enriched fraction (60% oleuropein) (a), standard oleuropein (b), and EGCG (c). The charts show means ± s.d. of percent MTT-formazan absorbance, logistic regression lines, and IC_50_ values expressed as *μ*g/mL (95% CI). Concentrations are normalized as the logarithm of *μ*g/mL.

The oleuropein-enriched fraction and standard oleuropein were separately used on REN cells at nominally equivalent oleuropein concentrations, in order to verify their antiproliferative activity. EGCG was used as positive control also in this case, since we had previously assessed its cytotoxicity on REN cells [24]. The cytotoxic effect of the oleuropein fraction (IC_50_ = 22 *μ*g/mL expressed as oleuropein) was slightly, but not significantly, stronger than that of standard oleuropein (IC_50_ = 25 *μ*g/mL). Both substances were slightly more cytotoxic than EGCG (IC_50_ = 33 *μ*g/mL) (Figure 2).

## 4. Discussion

The oleuropein yield in our enriched fraction is very close to the highest concentrations present in commercially available olive leaf extract formulations [25], suggesting its possible direct use in medicinal preparations. Despite a vast array of data on possible oleuropein cellular targets, our data about the effects on intracellular Ca^2+^ are new. The activity of oleuropein was slightly lower than that of the oleuropein-enriched fraction, possibly due to the occurrence in the fraction of other secoiridoid esters known to be present in olive leaves, such as demethyloleuropein, ligstroside, and oleuroside [26], allegedly coeluting with oleuropein in the HPLC separation.

We have previously found that low-voltage-activated T-type Ca^2+^ channels, specifically the isoform Cav3.2, mediate the Ca^2+^ rise induced in REN cells by the green tea polyphenol epigallocatechin-3-gallate [23]. We have also shown that Cav3.2 is responsible for Ca^2+^ spikes induced by epigallocatechin-3-gallate in MCF-7 breast cancer cells [20]. In the present study, an involvement of T-type Ca^2+^ channels is called into question also for the Ca^2+^ mobilizing effect of oleuropein by the use of Ca^2+^-free medium, indicating a Ca^2+^ entry process, and of the specific T-type Ca^2+^ channel blocker NNC-55-0396.

The stronger activity of hydroxytyrosol with respect to oleuropein and the inhibitory effect of NNC-55-0396 suggest that the hydroxytyrosol moiety could be the active site mediating the oleuropein effect on T-type Ca^2+^ channels. The almost complete inhibition exerted by NNC-55-0396 on hydroxytyrosol at lower dose, compared with the partial inhibition at higher dose, seems to indicate that the effect of olive phenolics mostly involves a T-type Ca^2+^ channel-dependent mechanism at low doses (≤10 *μ*M). At higher doses, these phenolics could instead act through a more complex mechanism, possibly including other Ca^2+^ mobilizing systems.

T-type Ca^2+^ channels have been linked to various kinds of epilepsy [27], while they are thought to play a role in tumor cell cycle progression and proliferation [28, 29]. Hence, much interest has been raised in their possible therapeutic targeting with more or less specific inhibitors [30, 31]. However, the blockage of these channels does not seem to be an exclusive strategy in antitumor therapy. Antiproliferative effects obtained with mibefradil and pimozide, two T-type Ca^2+^ channel inhibitors, have been partially ascribed to some unidentified mechanism [32]. Also, cell cycle progression needs a fine tuning of [Ca^2+^]_i_ oscillations, suggesting that intracellular Ca^2+^ dysregulation determining transient [Ca^2+^]_i_ rise may also interfere with tumor development, possibly leading to cell death [33]. Accordingly, pharmacological agents like olive phenolics, able to abnormally activate T-type Ca^2+^ channel activity, could be promising tools for the inhibition of cancerous cell growth.

In apparent contrast with the present data, it has been previously shown that an olive leaf extract exerts antagonistic effects on high-voltage-activated L-type Ca^2+^ channel, thereby inducing blood pressure lowering [34, 35]. A comparison between these previous reports and our study is difficult to make, owing to differences in both the extracts and the experimental models. However, such a complex of data advocates an intriguing scenario, entailing divergent effects of major olive phenolics on low-voltage and high-voltage Ca^2+^ channels.

More insight into the possibility of using the oleuropein fraction in antitumor treatment for mesothelioma is provided by our cytotoxicity tests, indicating that the antiproliferative activity of the oleuropein fraction essentially depends on its major constituent oleuropein. The United States National Cancer Institute plant screening program has established that a plant extract can be deemed to have a cytotoxic effect if IC_50_ for incubations of 48–72 h is 20 *μ*g/mL or lower [36]. Our oleuropein-enriched fraction has an oleuropein content of 60% and a cytotoxicity IC_50_ of 22 (17–29) *μ*g/mL in terms of oleuropein. We can then estimate IC_50_ of 36.7 (28.3–48.3) *μ*g/mL for the oleuropein-enriched fraction as a whole. Hence, our oleuropein fraction is close to the cytotoxicity threshold established by NCI and could therefore be appropriate for the development of synergistic treatments allowing reducing the doses of highly toxic conventional drugs, as previously suggested for EGCG [1]. Such an approach could overcome critical drawbacks in the treatment of chemoresistant tumors like malignant mesothelioma [37].

There is no established standard dose of oleuropein for humans, but in acute toxicity studies, oleuropein and hydroxytyrosol have shown no lethality or adverse effects in mice up to doses of 1000 and 2000 mg/kg, respectively. In addition, studies conducted on humans with olive extract or its polyphenolics have shown no undesired effects [38, 39]. Pharmacokinetics of oral oleuropein and its metabolite hydroxytyrosol in human plasma indicates peaks of about 0.004 *μ*g/mL (0.01 *μ*M) for oleuropein, while its main metabolite hydroxytyrosol can reach values of about 0.15 *μ*g/mL (1 *μ*M) [40]. These values are close to the effective concentrations observed in our experiments for hydroxytyrosol, but intravenous delivery of oleuropein should allow reaching much higher blood peaks. Hence, pharmacokinetics data indicate that plasma concentrations compatible with dysregulating effects on tumor cell Ca^2+^ could be easily achieved upon clinical administrations of oleuropein, followed by its conversion into the more effective metabolite hydroxytyrosol.

## 5. Conclusions

We have shown a method to obtain oleuropein-enriched olive leaf extract fractions that could be of industrial interest, because extraction is generally competitive with chemical synthesis for complex molecules like oleuropein. In addition, we have highlighted a novel mechanism of action for oleuropein and its metabolite hydroxytyrosol, consisting in a dysregulation of Ca^2+^ dynamics occurring via T-type Ca^2+^ channels. This kind of effect suggests a possible use of the oleuropein fraction from olive leaves in the treatment of mesothelioma.

## Figures and Tables

**Table 1:** sMRM NI transitions (Q1 and Q3 masses) and mass spectrometry parameters. The two most sensitive transitions for each species were monitored.

Analytes	Q1 (*m*/*z*)	Q3 (*m*/*z*)	*t*_*R*_ (min)	DP (V)	EP (V)	CEP (V)	CE (V)	CXP (V)
Hydroxytyrosol	153	123/93	3.4	−34	−75	−17.32	−20/−31	−1.08/−1.35
Oleuropein	539	275/307	7.9	−40	−5	−31.61	−28/−29	−1.89/−2.12

*t*_*R*_: retention time; DP: Declustering Potential; EP: Entrance Potential; CEP: Collision cell Entrance Potential; CE: Collision Energy; CXP: Collision cell eXit Potential.

**Table 2:** Percent content (w/w) of oleuropein and hydroxytyrosol in different olive leaf extracts.

	RE	SE	EF
Oleuropein	21.4 ± 0.3	43.2 ± 1.4	60.1 ± 0.3
Hydroxytyrosol	0.37 ± 0.04	0.91 ± 0.03	n.d.

Data are means ± s.d. of three independent measures. RE: raw extract; SE: subextract; EF: oleuropein-enriched fraction; n.d.: not detected.

## References

[1] Volta V., Ranzato E., Martinotti S. (2013). Preclinical demonstration of synergistic Active Nutrients/Drug (AND) combination as a potential treatment for malignant pleural mesothelioma. *PLoS ONE*.

[2] Diez C. M., Trujillo I., Martinez-Urdiroz N. (2015). Olive domestication and diversification in the Mediterranean basin. *The New Phytologist*.

[3] Alarcón de la Lastra C., Barranco M. D., Motilva V., Herrerías J. M. (2001). Mediterranean diet and health: biological importance of olive oil. *Current Pharmaceutical Design*.

[4] El S. N., Karakaya S. (2009). Olive tree (*Olea europaea*) leaves: potential beneficial effects on human health. *Nutrition Reviews*.

[5] Delgado-Pertíñez M., Chesson A., Provan G. J., Garrido A., Gómez-Cabrera A. (1998). Effect of different drying systems for the conservation of olive leaves on their nutritive value for ruminants. *Animal Research*.

[6] Garcia-Maraver A., Salvachúa D., Martínez M. J., Diaz L. F., Zamorano M. (2013). Analysis of the relation between the cellulose, hemicellulose and lignin content and the thermal behavior of residual biomass from olive trees. *Waste Management*.

[7] Savournin C., Baghdikian B., Elias R., Dargouth-Kesraoui F., Boukef K., Balansard G. (2001). Rapid high-performance liquid chromatography analysis for the quantitative determination of oleuropein in *Olea europaea* leaves. *Journal of Agricultural and Food Chemistry*.

[8] Ansari M., Kazemipour M., Fathi S. (2011). Development of a simple green extraction procedure and HPLC method for determination of oleuropein in olive leaf extract applied to a multi-source comparative study. *Journal of the Iranian Chemical Society*.

[9] Ryan D., Antolovich M., Prenzler P., Robards K., Lavee S. (2002). Biotransformations of phenolic compounds in *Olea europaea* L.. *Scientia Horticulturae*.

[10] Omar S. H. (2010). Oleuropein in olive and its pharmacological effects. *Scientia Pharmaceutica*.

[11] Bulotta S., Celano M., Lepore S. M., Montalcini T., Pujia A., Russo D. (2014). Beneficial effects of the olive oil phenolic components oleuropein and hydroxytyrosol: focus on protection against cardiovascular and metabolic diseases. *Journal of Translational Medicine*.

[12] Durlu-Özkaya F., Özkaya M. T. (2011). Oleuropein using as an additive for feed and products used for humans. *Journal of Food Processing & Technology*.

[13] Barbaro B., Toietta G., Maggio R. (2014). Effects of the olive-derived polyphenol oleuropein on human health. *International Journal of Molecular Sciences*.

[14] Granados-Principal S., Quiles J. L., Ramirez-Tortosa C. L., Sanchez-Rovira P., Ramirez-Tortosa M. C. (2010). Hydroxytyrosol: from laboratory investigations to future clinical trials. *Nutrition Reviews*.

[15] Bernini R., Merendino N., Romani A., Velotti F. (2013). Naturally occurring hydroxytyrosol: synthesis and anticancer potential. *Current Medicinal Chemistry*.

[16] Lee O.-H., Lee B.-Y. (2010). Antioxidant and antimicrobial activities of individual and combined phenolics in *Olea europaea* leaf extract. *Bioresource Technology*.

[17] Bracci T., Sebastiani L., Busconi M., Fogher C., Belaj A., Trujillo I. (2009). SSR markers reveal the uniqueness of olive cultivars from the Italian region of Liguria. *Scientia Horticulturae*.

[18] Mazzolini M., Traverso S., Marchetti C. (2001). Multiple pathways of Pb^2+^ permeation in rat cerebellar granule neurones. *Journal of Neurochemistry*.

[19] Grynkiewicz G., Poenie M., Tsien R. Y. (1985). A new generation of Ca^2+^ indicators with greatly improved fluorescence properties. *Journal of Biological Chemistry*.

[20] Ranzato E., Magnelli V., Martinotti S. (2014). Epigallocatechin-3-gallate elicits Ca^2+^ spike in MCF-7 breast cancer cells: essential role of Cav3.2 channels. *Cell Calcium*.

[21] Li M., Hansen J. B., Huang L., Keyser B. M., Taylor J. T. (2005). Towards selective antagonists of T-type calcium channels: design, characterization and potential applications of NNC 55-0396. *Cardiovascular Drug Reviews*.

[22] Palmerini C. A., Carlini E., Saccardi C., Servili M., Montedoro G., Arienti G. (2005). Activity of olive oil phenols on lymphomonocyte cytosolic calcium. *The Journal of Nutritional Biochemistry*.

[23] Ranzato E., Martinotti S., Magnelli V. (2012). Epigallocatechin-3-gallate induces mesothelioma cell death via H_2_O_2_-dependent T-type Ca^2+^ channel opening. *Journal of Cellular and Molecular Medicine*.

[24] Martinotti S., Ranzato E., Burlando B. (2011). In vitro screening of synergistic ascorbate-drug combinations for the treatment of malignant mesothelioma. *Toxicology in Vitro*.

[25] Kremastinos D. T. (2008). Olive and oleuropein. *Hellenic Journal of Cardiology*.

[26] Wang Y., Wang S. Q., Cui W. H., He J. J., Wang Z. F., Yang X. L. (2013). Olive leaf extract inhibits lead poisoning-induced brain injury. *Neural Regeneration Research*.

[27] Tringham E., Powell K. L., Cain S. M. (2012). T-type calcium channel blockers that attenuate thalamic burst firing and suppress absence seizures. *Science translational medicine*.

[28] Panner A., Cribbs L. L., Zainelli G. M., Origitano T. C., Singh S., Wurster R. D. (2005). Variation of T-type calcium channel protein expression affects cell division of cultured tumor cells. *Cell Calcium*.

[29] Taylor J. T., Zeng X.-B., Pottle J. E. (2008). Calcium signaling and T-type calcium channels in cancer cell cycling. *World Journal of Gastroenterology*.

[30] Li W., Zhang S.-L., Wang N., Zhang B.-B., Li M. (2011). Blockade of T-type Ca^2+^ channels inhibits human ovarian cancer cell proliferation. *Cancer Investigation*.

[31] Loughlin K. R. (2014). Calcium channel blockers and prostate cancer. *Urologic Oncology*.

[32] Bertolesi G. E., Shi C., Elbaum L. (2002). The Ca^2+^ channel antagonists mibefradil and pimozide inhibit cell growth via different cytotoxic mechanisms. *Molecular Pharmacology*.

[33] Monteith G. R., McAndrew D., Faddy H. M., Roberts-Thomson S. J. (2007). Calcium and cancer: targeting Ca^2+^ transport. *Nature Reviews Cancer*.

[34] Scheffler A., Rauwald H. W., Kampa B., Mann U., Mohr F. W., Dhein S. (2008). Olea europaea leaf extract exerts L-type Ca^2+^ channel antagonistic effects. *Journal of Ethnopharmacology*.

[35] Gilani A. H., Khan A.-U., Shah A. J., Connor J., Jabeen Q. (2005). Blood pressure lowering effect of olive is mediated through calcium channel blockade. *International Journal of Food Sciences and Nutrition*.

[36] Geran R. I., Greenberg N. H., Macdonald M. M., Shumacher A. M., Abbott B. J. (1972). Protocols for screening chemical agents and natural products against animal tumors and other biological systems. *Cancer Chemotherapy Reports Part 3*.

[37] Suganuma M., Saha A., Fujiki H. (2011). New cancer treatment strategy using combination of green tea catechins and anticancer drugs. *Cancer Science*.

[38] Visioli F., Galli C. (2001). Phenolics from olive oil and its waste products. Biological activities in in vitro and in vivo studies. *World Review of Nutrition and Dietetics*.

[39] González P., Florido F., Sáenz de San Pedro B., de la Torre F., Rico P., Martín S. (2002). Immunotherapy with an extract of *Olea europaea* quantified in mass units: evaluation of the safety and efficacy after one year of treatment. *Journal of Investigational Allergology and Clinical Immunology*.

[40] de Bock M., Thorstensen E. B., Derraik J. G. B., Henderson H. V., Hofman P. L., Cutfield W. S. (2013). Human absorption and metabolism of oleuropein and hydroxytyrosol ingested as olive (*Olea europaea* L.) leaf extract. *Molecular Nutrition and Food Research*.

